# Recent Advances in Membrane Filtration and Purification Technologies

**DOI:** 10.3390/membranes16030078

**Published:** 2026-02-24

**Authors:** Ahmed M. Khalil

**Affiliations:** Photochemistry Department, National Research Centre, Dokki, Giza 12622, Egypt; akhalil75@yahoo.com

Membrane technologies comprise a broad range of materials and structural designs tailored for specific separation applications, including gas separation [1], water purification, wastewater treatment, and energy-related processes. Based on material composition, membranes can be broadly classified as polymeric, inorganic (ceramic and metallic) or hybrid membranes, each offering distinct advantages and limitations. Polymeric membranes dominate large-scale applications due to their low cost, ease of fabrication, and tunable physicochemical properties, making them widely used in water remediation processes such as microfiltration, ultrafiltration, nanofiltration, desalination and reverse osmosis. In contrast, inorganic membranes, comprising ceramic and metallic membranes, exhibit superior thermal, chemical, and mechanical stability. Hence, they are suitable for harsh operating environments. Hybrid systems, such as mixed-matrix membranes that combine polymers with inorganic fillers, have emerged as promising solutions to overcome the permeability and selectivity trade-off while expanding the applicability of membrane technologies across diverse industrial sectors [2,3]. In application-specific contexts, membrane design is closely aligned with separation mechanisms and operating requirements. For water remediation, polymeric and polymer-based composite membranes are predominantly employed to remove suspended solids, organic pollutants, dyes and heavy metals through size exclusion, charge-based repulsion and affinity-driven interactions. Surface modification and nanocomposite strategies are increasingly used to enhance fouling resistance and contaminant selectivity. Conversely, hydrogen separation membranes demand materials with exceptional hydrogen permeability and selectivity, leading to the historical dominance of dense metallic membranes, particularly palladium-based alloys, as well as the growing interest in ceramic proton-conducting membranes and advanced alloy systems. Recent research efforts are converging toward multifunctional membrane platforms that integrate high selectivity, durability, and cost effectiveness, enabling membranes to play a critical role in both clean energy production and sustainable water treatment, two of the most pressing technological challenges of the modern era [4].

Hydrogen separation membranes are critical components in clean energy systems, particularly for purifying hydrogen produced from steam reforming, coal gasification, methane pyrolysis, and water electrolysis processes. These membranes enable selective permeation of hydrogen over other gases (e.g., CO_2_, N_2_, CH_4_), offering an energy-efficient alternative to conventional methods such as cryogenic distillation and pressure swing adsorption. While dense metallic-based alloys have historically dominated due to their high solubility and selectivity for hydrogen, they face challenges including high cost and susceptibility to poisoning [5]. Consequently, polymeric membranes, mixed-matrix membranes (MMMs) that integrate inorganic fillers into polymer matrices, and other advanced gas-separation materials are being developed to balance permeability and selectivity with lower fabrication costs and improved mechanical properties. These materials are engineered to enhance H_2_ selectivity while maintaining robust performance under relevant operating conditions, positioning membrane technology as a key enabler in hydrogen purification for sustainable energy systems [6].

The increasing scarcity of clean water resources has emerged as one of the most critical global challenges of the twenty-first century, driven by rapid industrialization, urban expansion, and population growth. Industrial effluents containing organic pollutants, synthetic dyes, and toxic heavy metals are major contributors to water contamination, posing severe risks to ecosystems and human health. Conventional water treatment technologies, such as coagulation, adsorption, and biological treatment, often suffer from limitations related to efficiency, selectivity, and operational cost. In this context, membrane-based separation processes have gained significant attention as promising and sustainable alternatives for water remediation [7,8].

Composite membranes, in particular, have become principal to modern water treatment technologies due to their tunable physicochemical properties, scalability, and cost-effectiveness. Moreover, polymeric membranes function as selective barriers that allow water molecules to pass while rejecting contaminants based on size exclusion, charge interactions or affinity-based mechanisms. Compared with ceramic and metallic membranes, polymeric membranes offer advantages such as flexibility in fabrication, lower energy requirements, and ease of surface modification. As a result, they have been extensively investigated for applications in microfiltration, ultrafiltration, nanofiltration, and reverse osmosis processes [9,10].

Water pollutants encompass a wide range of contaminants, including suspended solids, organic compounds, dyes, pharmaceuticals, and heavy metals. Many of these substances are persistent, non-biodegradable, and capable of bioaccumulation, leading to long-term environmental consequences. Polymeric membranes provide an effective platform for addressing this complexity, as their separation performance can be tailored by controlling pore size, surface charge, hydrophilicity and membrane morphology. This adaptability makes them particularly suitable for treating industrial wastewater streams with variable compositions. Synthetic dyes represent a major class of water pollutants, especially in effluents from textile, leather, paper, and dyeing industries. These compounds are typically characterized by complex aromatic structures, high solubility, and resistance to degradation. Even at low concentrations, dyes impart intense color to water bodies, reducing light penetration and disrupting aquatic ecosystems. Polymeric membranes, especially nanofiltration and ultrafiltration membranes, have demonstrated high dye rejection efficiencies through size exclusion and electrostatic interactions, offering an effective means of decolorization and pollutant removal. In addition to dyes, heavy metals such as lead, cadmium, chromium, mercury, and arsenic are among the most hazardous water contaminants due to their toxicity, persistence, and tendency to accumulate in living organisms. Industrial activities including mining, electroplating, battery manufacturing, and metal finishing are primary sources of heavy metal pollution. Polymeric membranes can effectively remove heavy metal ions by mechanisms such as complexation with functional groups and adsorption onto modified membrane surfaces [11,12]. These capabilities have positioned membrane processes as key technologies for heavy metal remediation. The performance of polymeric membranes in water remediation is strongly influenced by the choice of polymer material. Commonly used polymers include polysulfone, polyethersulfone, polyvinylidene fluoride, cellulose acetate, and polyamide. Each material offers distinct advantages in terms of chemical resistance, mechanical strength, and permeability. However, challenges such as membrane fouling, limited selectivity, and trade-offs between permeability and rejection remain significant obstacles to widespread application.

Membrane fouling, caused by the accumulation of organic matter, dyes, microorganisms, and inorganic salts on the membrane surface or within pores, is a major limitation in polymeric membrane processes. Fouling leads to reduced flux, increased energy consumption, and shortened membrane lifespan. To mitigate these issues, extensive research has focused on membrane surface modification strategies, including blending, coating, grafting, and incorporation of hydrophilic or antimicrobial additives. These approaches aim to enhance fouling resistance while maintaining or improving separation performance. Recent advances in polymer chemistry and nanotechnology have enabled the development of advanced polymeric membranes with enhanced functionality. The incorporation of nanoparticles, such as metal oxides, carbon-based nanomaterials, and zeolites, has led to the emergence of mixed-matrix and nanocomposite membranes. These materials can improve permeability, selectivity, mechanical stability, and contaminant affinity. Such innovations have shown particular promise in the removal of dyes and heavy metals from complex wastewater matrices [13,14]. Another important direction in membrane research is the design of membranes with tailored surface charge and functional groups. Functionalization with chelating agents, sulfonic groups, amines, or carboxyl groups can significantly enhance the binding and rejection of metal ions and charged dye molecules. These chemically modified membranes enable more selective and efficient removal processes, reducing the need for additional treatment steps and improving overall system sustainability. From a process perspective, membranes are often integrated into hybrid treatment systems to overcome the limitations of single-stage technologies. Coupling membrane filtration with adsorption, advanced oxidation processes or biological treatment can lead to synergistic effects, improving contaminant removal efficiency and operational stability [15,16]. Such integrated approaches are increasingly being explored for industrial wastewater treatment, where complex pollutant mixtures require multifunctional remediation strategies. Despite significant progress, several challenges must still be addressed to fully realize the potential of membranes in water remediation. Issues related to the long-term stability, fouling control, membrane recyclability, and environmental impact of membrane fabrication remain critical. Furthermore, the economic feasibility of large-scale applications depends on reducing material costs and energy consumption while maintaining high performance. Addressing these challenges requires interdisciplinary research combining materials science, chemical engineering, and environmental science.

Membranes have emerged as versatile and effective tools for the remediation of water contaminated with pollutants, dyes, and heavy metals. Their tunable properties, compatibility with advanced modification techniques, and suitability for integration into hybrid systems make them highly attractive for sustainable water treatment applications. Continued innovation in membrane materials and process design is expected to further enhance their performance and expand their role in addressing global water pollution challenges. This Special Issue (SI) aims to provide a comprehensive overview of recent developments and future perspectives in polymeric membrane technologies for water remediation membrane filtration and purification technologies. Membrane-based separation technologies continue to play a vital role in addressing global challenges related to water treatment, environmental protection, and sustainable energy systems. Ongoing advances in membrane materials, fabrication methods and process design are essential to improve separation efficiency, selectivity and long-term operational stability. This Special Issue, entitled “*Recent Advances in Membrane Filtration and Purification Technologies*”, brings together eight original research articles that collectively reflect recent progress and emerging trends in membrane science and engineering.

Several contributions focus on pressure-driven membrane processes for water purification. Lim et al. (contribution 1) investigated the removal of phthalates using nanofiltration and reverse osmosis membranes, emphasizing the influence of membrane surface properties on micropollutant rejection. Their findings provide important insights into membrane–solute interactions and offer guidance for the selection of membranes in advanced water treatment applications. In a relevant study, El Sayed et al. (contribution 2) evaluated a reverse osmosis-based zero liquid discharge system for the desalination of agricultural drainage water. By combining performance analysis with environmental and economic considerations, this work demonstrates the potential of membrane-based strategies to enhance water recovery while minimizing brine disposal. Advances in osmotically driven and energy-related membrane processes are also addressed. Moon and Kang (contribution 3) examined the effect of anion selection in tetrabutylphosphonium-based draw solutes on forward osmosis performance. Their results highlight the critical role of draw-solute chemistry in determining osmotic pressure and water flux, contributing to the optimization of forward osmosis systems.

In the context of hydrogen separation, Kashkarov et al. (contribution 4) examined Nb-Ni-Ti-Zr-Co high-entropy alloys as membrane materials. By correlating alloy microstructure with hydrogen permeability, the authors demonstrate the potential of high-entropy alloys for membrane applications in clean energy technologies.

A significant portion of this SI is devoted to the development of functional and nanocomposite membranes for pollutant removal. Alrebdi et al. (contribution 5) reported the fabrication of Ag-doped ZnO/MWCNT nanocomposite membranes via pulsed laser deposition, showing enhanced photocatalytic degradation of Rhodamine B. Similarly, Alrebdi et al. (contribution 6) developed Ag/ZnO thin-film nanocomposite membranes capable of efficiently degrading 4-nitrophenol, illustrating the advantages of integrating catalytic activity within membrane structures. In another contribution, Alkallas et al. (contribution 7) presented poly(vinyl alcohol)/Al_2_O_3_ nanocomposite films prepared by pulsed laser ablation in liquid, demonstrating effective removal of Ni(II) ions and highlighting the potential of nanocomposite membranes for heavy-metal remediation.

The Special Issue is brought to completion by a study on electrospun biodegradable composite membranes based on polyhydroxybutyrate and poly(vinyl formate) loaded with protonated montmorillonite performed by Penchev et al. (contribution 8). This work investigates the adsorption of organic dyes, including methylene blue and Congo red, and provides kinetic and isotherm analyses to elucidate the adsorption mechanisms. The results emphasize the role of fibrous membrane morphology and clay incorporation in enhancing dye removal performance, while also addressing sustainability considerations through the use of biodegradable polymers.

In summary, the eight articles collected within this SI highlight recent advances in membrane filtration and purification technologies, with particular emphasis on material innovation, multifunctional membrane design and application-oriented solutions. The contributions demonstrate the growing importance of membranes in water treatment, energy-related separations and environmental remediation. This Special Issue aims to serve as a valuable reference and stimulate further research in the field. As this SI draws to a close, it is clear that membrane science continues to advance at a remarkable pace, driven by the urgent need for more sustainable and effective separation technologies. The assembled contributions underscore significant progress in a broad range of membrane processes from traditional filtration and reverse osmosis to innovative hybrid and multifunctional systems. They highlight both fundamental insights and practical applications across water treatment, desalination, and industrial purification challenges. These studies reflect the community’s commitment to not only deepening our understanding of membrane behavior but also addressing real-world issues such as energy use, fouling, and environmental impact. A consistent theme across the published works is the exploration of novel membrane materials and tailored structural designs that improve performance while mitigating long-standing obstacles like fouling and selectivity loss. Research articles in this issue have showcased material innovation, whether through hybrid composites, advanced surface functionalization, or engineered pore structures. They can deliver enhanced flux, selectivity, and durability under varied operational conditions. These advancements point toward a future in which membranes are not mere passive barriers but engineered interfaces capable of responsive and robust separation performance. The practical implications of these technological breakthroughs are equally noteworthy. Whether applied to wastewater recycling, oil/water emulsion separation, or protein purification, the cutting-edge studies presented here demonstrate the tangible benefits of membrane technology in tackling environmental and industrial purification tasks. Moreover, many contributions emphasize the balance between high separation efficacy and cost-effective design, aligning scientific innovation with the broader goals of sustainability and scalability in membrane applications.

Looking forward, the field is poised to make even greater strides by embracing interdisciplinary approaches that connect materials science, process engineering, and systems analysis. Continued collaboration among researchers, industry stakeholders, and policymakers will be essential to translate laboratory success into large-scale deployment that addresses global water scarcity, pollution, and resource recovery challenges. As this Special Issue has shown, the collective effort of the membrane community holds promise for not only refining existing technologies but also inspiring transformative solutions that contribute to a more sustainable future.
